# Additive Manufacturing of Biodegradable Hemp-Reinforced Polybutylene Succinate (PBS) and Its Mechanical Characterization

**DOI:** 10.3390/polym15102271

**Published:** 2023-05-11

**Authors:** Antonia Dönitz, Anton Köllner, Tim Richter, Oliver Löschke, Dietmar Auhl, Christina Völlmecke

**Affiliations:** 1 Stability and Failure of Functionally Optimized Structures, Institute of Mechanics, Technische Universität Berlin, Einsteinufer 5, 10587 Berlin, Germany; 2 Polymer Materials and Technologies, Institute of Material Science and Technology, Technische Universität Berlin, Ernst-Reuter-Platz 1, 10587 Berlin, Germany

**Keywords:** additive manufacturing, FFF printing, printing parameters, biopolymer, natural fibre reinforcement, experiment, stiffness, tensile strength, hemp, PBS

## Abstract

The additive manufacturing of natural fibre-reinforced polymers is a pivotal method in developing sustainable engineering solutions. Using the fused filament fabrication method, the current study investigates the additive manufacturing of hemp-reinforced polybutylene succinate (PBS) alongside its mechanical characterization. Two types of hemp reinforcement are considered: short fibres (max. length smaller than 2 mm) and long fibres (max. length smaller than 10 mm), which are compared against non-reinforced (pure) PBS. A detailed analysis is performed regarding the determination of suitable 3D printing parameters (overlap, temperature, nozzle diameter). In a comprehensive experimental study, additionally to general analyses regarding the influence of hemp reinforcement on the mechanical behaviour, the effect of printing parameters is determined and discussed. Introducing an overlap in the additive manufacturing of the specimens results in improved mechanical performance. The study highlights that the Young’s modulus of PBS can be improved by 63% by introducing hemp fibres in conjunction with overlap. In contrast, hemp fibre reinforcement reduces the tensile strength of PBS, while this effect is less pronounced considering overlap in the additive manufacturing process.

## 1. Introduction

Additive manufacturing enables more efficient and sustainable production processes. During production, only the amount of material is used that is also required to manufacture the printed end-product [1]. Thus, no or only little unnecessary resource consumption remains for disposal. In addition to a more sustainable production, renewable and biodegradable raw materials from bio-based sources may also be used for various products in order to achieve sustainable production [2,3]. The additive manufacturing technology fused filament fabrication (FFF), a process in which a component is built up layer by layer through the deposition of material based on digital 3D design data [4,5], can be regarded as the most prominent additive manufacturing process. However, further technologies based on the deposition of fused thermoplastic polymers exist, such as laser-assisted FFF [6], electrohydrodynamic direct-writing [7] and direct sound printing [8].

In the FFF method, some issues related to both processing and end-use properties must be considered, which are connected to flow behaviour, layer bonding, or porosity, for example. Other challenges such as nozzle clogging [9] and satellite droplets [10] have to be also considered during manufacturing. Therefore, special attention must be given to consider and avoid such major obstacles attached to these flaws [11,12,13,14,15], which becomes particularly relevant considering the additive manufacturing of natural fibre or particle-reinforced polymers as presented in this work. Many studies focus on reinforcing bio-based polymers (e.g., polylactic acid, polybutylene succinate) with natural fibres by conventional manufacturing processes, e.g., see [16,17,18]. For the FFF method, attempts at integrating natural fibre reinforcements have been made, where studies mainly considered the polymer polylactic acid [2,19]. Polybutylene succinate co-adipate (PBSA) in combination with bio-fillers (Typha stem powder) is considered in [20].

Polybutylene succinate (PBS) is a semi-crystalline thermoplastic polyester that can be produced from either fossil or renewable resources. It is produced by poly-condensation of succinic acid and 1,4-butanediol, which can be obtained from glucose and sucrose through fermentation. In the latter case, it is not only biodegradable via composting, but also bio-based [21]. PBS can break down into, water, carbon dioxide, and biomass with the help of soil microorganisms. It has high heat resistance and biocompatibility with natural fibres due to its semi-crystalline nature and chemical structure similar to plant-based fibres. PBS has a low glass-transition temperature between about −20 and −40 °C and a high melting temperature of about 115 °C [21,22,23]. PBS has, unlike PLA, excellent impact strength and heat resistance as well as high elongation at breaking [17,24]. It is suitable for thermoforming and can be blended with other biopolymers [17,24]. It has various applications in packaging, such as films, trays, boxes, compostable cutlery, as well as in medical, forestry and fishery products to reduce plastic waste.

Hemp is a natural material that has been extensively used throughout human history; for example, as a source material in ropes and sail canvas. Hemp fibres are generally obtained from the bast part of the cannabis plant and possess high mechanical stiffness from about 17 to 66 GPa [25], see Table 1. The advantage of hemp as a reinforcement of PBS for biomaterial composites is the environmental friendliness and biodegradability in contrast to glass-fibre composites. In this context, hemp also increases the composting rate compared to regular PBS [26]. This biocomposite is deemed to offer not only a higher tensile modulus at ambient conditions, but also higher heat-deflection temperatures at elevated temperatures of 100 °C [27,28,29]. However, it is known from fibre composites that effective fibre length plays a crucial role. Longer hemp fibre particles are thought to have larger friction forces between long hemp particles and the PBS matrix and can be greater than between short hemp fibres or powder particles and the PBS matrix. It should also be noted that PBS particles interact with highly lignified fibres such as hemp and flax compared to other plant fibres with lower lignin content as determined by AFM-based force spectroscopy studies [30].

The objective of this study is to explore the additive manufacturing of hemp-reinforced PBS alongside its mechanical characterization. To the authors knowledge, for the FFF method, this material combination has not been investigated yet. Requirements and parameters to facilitate the 3D printing of hemp-reinforced PBS filaments are presented and discussed. The study therefore provides a blueprint for future studies aiming at manufacturing hemp-reinforced PBS. The effect of hemp reinforcement on the mechanical behaviour of the composite hemp-PBS is investigated by an experimental study. In addition to a general analysis of the mechanical behaviour, the effect of printing parameters such as overlap, printing temperature and nozzle diameter on the mechanical behaviour is investigated.

## 2. Materials and Additively Manufactured Specimens

### 2.1. Raw Materials

#### 2.1.1. PBS

Polybutylene succinate (PBS) was obtained from Mitsubishi Chemical Performance Polymers (MCPP, Düsseldorf, Germany) as food grade (FZ71 PM) [23]. For PBS, depending on the material grade, the tensile strength ranges from 20 to 40 MPa [22,23,24]. The elongation at break is also highly affected by the material grade and ranges from 15 to 170% [23,24], while the melting point is around 109–115 °C [22,23], see Table 1. Typically, the Young’s modulus of PBS is between 300 and 700 MPa depending on the crystallinity, see [22,31].

#### 2.1.2. Hemp (Particles)

**Figure 1:**
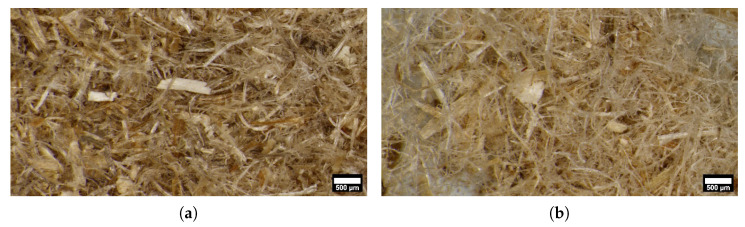
Hemp particles used in the present study; (**a**) short fibres (SF)–maximum length ≤ 2 mm (**b**) long fibres (LF)–maximum length ≤ 10 mm.

Two types of hemp were purchased as insulation material grade (SF: micro, LF: long particle) from a local provider for building and construction (Kenter Gbr., Frauenzimmer, Germany). According to micro-imaging analysis the hemp material is mostly composed of fibres together with a few particles, as can be seen in Figure 1. Material parameters of hemp fibres are provided in Table 1.

### 2.2. Reinforced Material—Filaments

Hemp particles with 5%-wt. and PBS granules were blended in a twin-screw extruder (Leistritz, Germany) to form the composite material. The extrudate was pulled through a cooling bath and cut into pellets afterwards. The pellets were dried in a vacuum oven for 60 h to significantly reduce the moisture content before processing via a single-screw (Axon, Sweden) extruder to produce a homogeneous filament with a constant diameter of 1.8 mm. Pure PBS was processed directly in the single-screw extruder. The extrusion parameters used to manufacture the filaments are provided in Table 2.

### 2.3. Composite Material Properties

With the material parameters of PBS and hemp provided in Section 2.1.1 and Section 2.1.2, respectively, an upper bound for the tensile stiffness of the PBS-hemp composite (the manufactured specimens) can be estimated. Therefore, following [32], the relative fibre volume content φ is determined by

(1) $$\varphi =\frac{1}{1+\frac{1-\Delta }{\Delta }\left(\frac{\rho_{h}}{\rho_{p}}\right)},$$

where $\rho_{p}$ and $\rho_{h}$ are the densities of hemp and PBS, respectively, and Δ is the weight fraction of the hemp fibres in the filament (0.05). A fibre volume fraction of 4.6% is calculated.

Using the mixing rule within the classical laminate theory [32], the upper bound of the tensile stiffness *E* can be calculated by

(2) $$E=\varphi E_{h}+\left(1-\varphi \right)E_{p},$$

with $E_{h}$ and $E_{p}$ being the Young’s modulus of hemp and PBS, respectively. Using the parameters provided in Section 2.1.1 and Section 2.1.2, an upper bound of 1520 MPa is calculated. It should be noted that in Equation (2), the assumptions of continuous and perfectly aligned fibres according to classical laminate theory is implemented for which the result obtained should be regarded as the upper bound for the hemp particle-reinforced PBS at hand with a volume fraction of 4.6%.

### 2.4. Specimen Production

**Figure 2:**
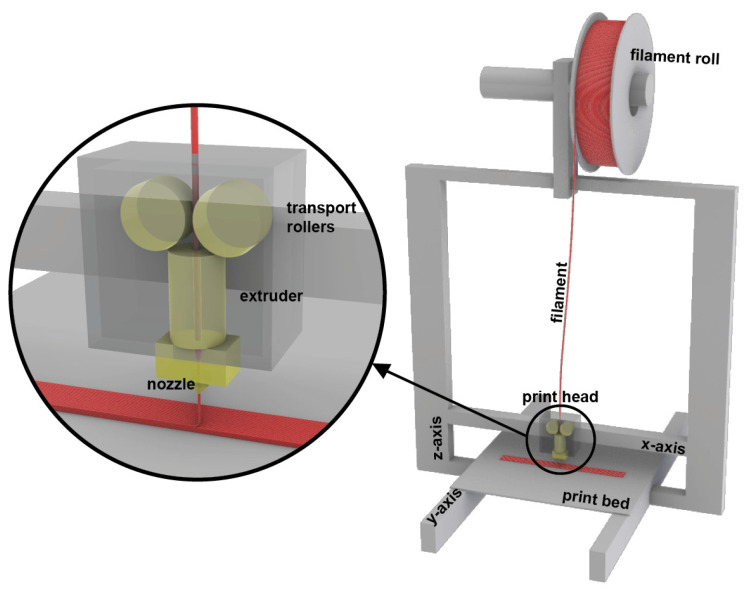
Schematic of FFF 3D printing process according to the Prusa i3MK3s.

In the following steps, the additive manufacturing method FFF will be employed to further process the composite polymer filament at hand into appropriate tensile test specimens. In this study, FFF printing is utilized as it is best suited for printing polymer particle composites. The technique offers geometric freedom and lightweight structure production. Particle- and fibre-reinforced materials can improve the mechanical performance through their structural benefits. However, hemp-reinforced PBS cannot easily be printed on all FFF 3D printers. Investigations into the behaviour of the composite material during printing may help in finding the optimal printer modifications to achieve reliable quality prints. In the FFF method, the print head operates on two of the three axes in the three-dimensional coordinate system. Specifically, in this study, the Prusa i3 MK3S+ (Prusa Research a.s., Prague, Czech Republic) was used (see Figure 2), the print head of which moves along the *x*-axis and *z*-axis. However, the *y*-axis movement, which is necessary for the complete three-dimensional printing process, is delegated to the print bed. Notably, other conventional printers have print beds that move on the *z*-axis, such as the Ultimaker3 extended. The filament is fed into the print head through transport rollers, which then undergoes heating in the extruder. Once melted, the material is extruded through the nozzle and applied onto the print bed in a series of layers. The process is visualised in Figure 2.

**Figure 3:**
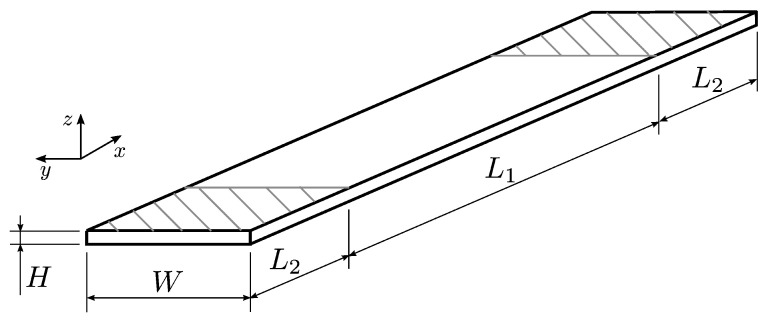
Schematic of the test specimens following ASTM D3039; cross-hatched areas ($L_{2}$) indicate the area of the tabs.

The experimental study comprises the following steps. First, the specimens are designed in accordance with the selected tensile test standard ASTM D3039 [33]. A schematic showing the dimensions of the test specimens is provided in Figure 3. The computer-aided 3D modelling software Rhinoceros 3D is used to create a model with the measurements $L_{1}=120$ mm, $L_{2}=30$ mm, W=15 mm and H=2 mm.

After evaluating the manufacturing of the preliminary specimens and the pretests, which are discussed in more detail in Section 3.1, the goal of the main test series is the analysis of the influence overlap, printing temperature and nozzle diameter have on the mechanical performance of the tensile specimens. The print parameters have been defined in the open-source slicer software Ultimaker Cura (version 5.2.1). The final print parameters are provided in Table 3.

**Figure 4:**
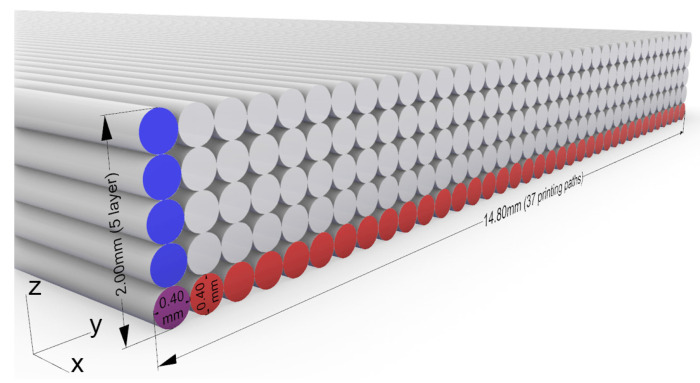
Detailed view of test specimen, number of layers and printing paths.

The layer height and width is 0.4 mm. With a height of 2 mm and a width of 15 mm for the specimen geometry, this results in 5 layers of 37 printing paths each. A detailed view of the specimen showing the effects of layer height and layer width on the print geometry can be found in Figure 4.

**Figure 5:**
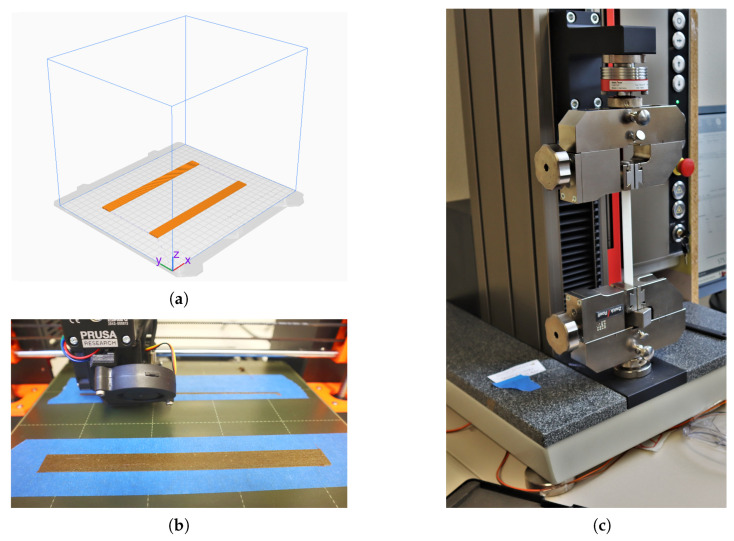
(**a**) Raster bed orientation (**b**) 3D printer Prusa i3 MK3S+ used for printing the specimens, (**c**) testing machine ZwickRoell Z2.5 used for testing the specimens.

All specimens have been additively manufactured using the aforementioned Prusa i3 MK3S+. Each PBS and PBS + 5% hemp particles tensile specimens have the dimensions 180 mm × 15 mm × 2 mm (length × width × thickness). Two samples were printed at once, with the 180 mm × 15 mm surface facing down on to the print bed, as shown in Figure 5a. Material tests have been conducted with the testing machine ZwickRoell Z2.5 (ZwickRoell GmbH & Co. KG August-Nagel-Straße 11, 89079 Ulm, Germany). The 3D printer and the testing machine are shown in Figure 5b,c, respectively.

Subsequently, the preliminary prints and tests as well as the main test series are described in detail.

## 3. Experimental Test Series

### 3.1. Pilot Investigations

**Figure 6:**
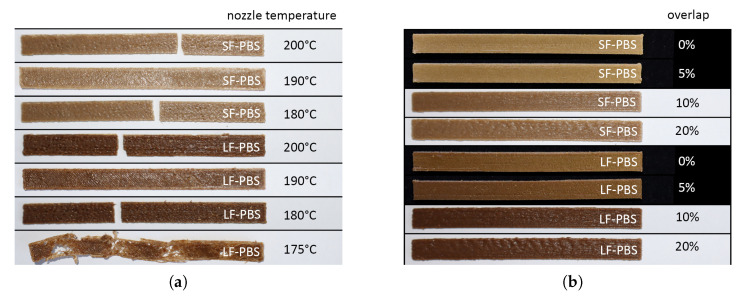
(**a**) First pre-tests printed with different nozzle temperatures: short fibre-reinforced PBS (SF-PBS) with 180, 190 and 200 °C printing temperature, long fibre-reinforced PBS (LF-PBS) with 180, 190 and 200 °C printing temperature and a failed print of long fibre-reinforced PBS at 175 °C. (**b**) Pre-tests printed with different overlap of the printing paths at a temperature of 180 °C: short fibre-reinforced PBS (SF-PBS) with 0, 5, 10 and 20% overlap, long fibre-reinforced PBS (LF-PBS) with with 0, 5, 10 and 20% overlap.

The aim of conducting preliminary prints and tests was to identify printing parameters that ensure the high-quality printability of specimens while providing the best possible mechanical characteristics. The printing parameters and the geometry of the specimens in the pilot investigations are provided in Table 4. First, the temperature range for facilitating the FFF printing of hemp fibre-reinforced PBS was determined. Prints at several temperatures were conducted, where a minimal printing temperature of 180 ${}^{{}^{\circ}}C$ was identified. Lower temperatures resulted in insufficient embedding of the hemp fibres with molten PBS, which is highlighted in Figure 6b (bottom specimen–printing temperature of 175 ${}^{{}^{\circ}}C$) showing specimens of the pilot investigations. The upper bound of possible printing temperatures was determined with the aid of thermogravimetric analysis of the hemp fibres. The analysis indicated that hemp remains stable up to approximately 220 ${}^{{}^{\circ}}C$, which therefore represents the upper bound for printing temperatures. However, printing temperatures over 200 ${}^{{}^{\circ}}C$ cannot be recommended since the printing in controlled dimensions becomes extremely cumbersome due to the low viscosity of PBS at such high temperatures. As a consequence, suitable printing temperatures range between 180 ${}^{{}^{\circ}}C$ and 200 ${}^{{}^{\circ}}C$.

**Figure 7:**
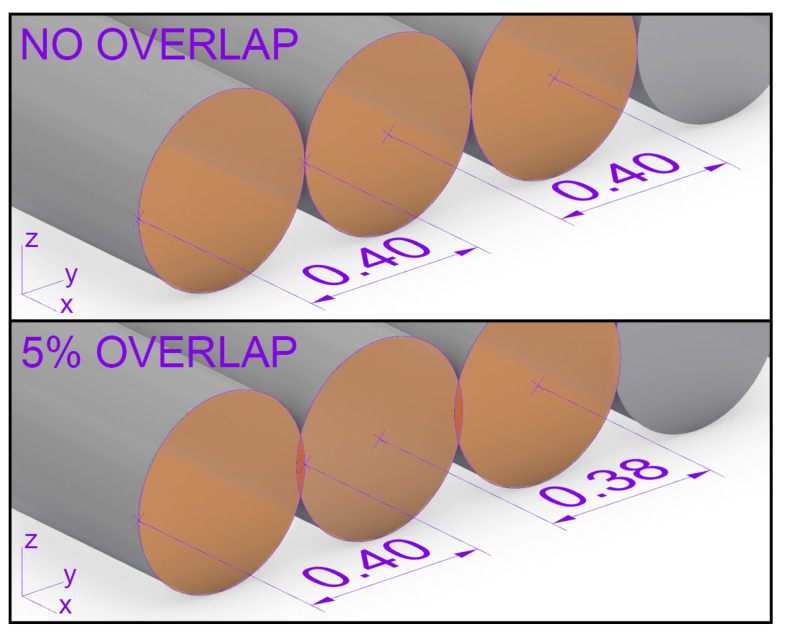
Theoretical overlap from slicing.

The implementation of overlap in the additive manufacturing process was also explored. The concept of overlap is visualized in Figure 7, achieved by manipulating the corresponding printing parameter infill line distance. Specimens with 0, 5, 10 and 20% overlap were manufactured as shown in Figure 6b. Note that the standard printing configuration does not consider overlaps. In pre-tests, a positive effect on the strength of the specimens was observed due to overlap. Using the concept of overlap results in a more homogenized material where single printing lines and layers appear to be better connected. A reduction in voids and gaps could also be identified by visual inspections using a microscopic device. However, any overlap larger than 5% has resulted in specimens suffering from inconsistent geometry due to uncontrollable outflow of material from the baseline geometry due to squeezing material in the pre-defined volume. Such misprints are also shown in Figure 6b.

In summary, the additive manufacturing of hemp fibre-reinforced PBS should consider printing temperatures in the range of 180 ${}^{{}^{\circ}}C$ to 200 ${}^{{}^{\circ}}C$ while overlaps of up to 5% may be used to improve the mechanical performance.

### 3.2. Main Test Series

**Figure 8:**
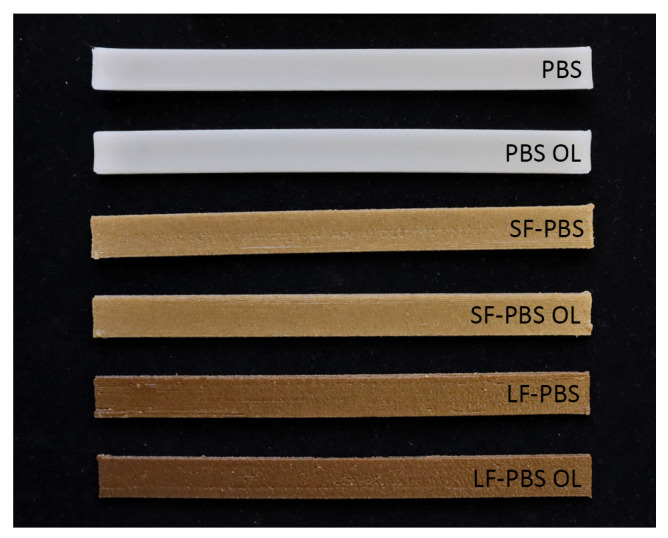
Overview of additively manufactured specimen types: ‘pure PBS’ (PBS, PBS OL), short fibre-reinforced PBS (SF-PBS, SF-PBS OL), long fibre-reinforced PBS (LF-PBS, LF-PBS OL), OL–overlap of 5% used in printing, specimens printed at $180{\;}^{{}^{\circ}}$C (specimens printed at a higher temperature of 200 ${}^{{}^{\circ}}C$ (‘HT’) are not shown).

Three general specimen types were investigated in the main test series: pure PBS, short fibre-reinforced PBS and long fibre-reinforced PBS, which are subsequently denoted by ‘PBS’, ‘SF-PBS’ and ‘LF-PBS’. Based on the findings obtained from the preliminary prints and tests, the main test study was designed in two parts. First, the effect of overlap was investigated by comparing material tests conducted with all specimens types considering no overlap and an overlap of 5% denoted by ‘OL’ subsequently (test study A). In the second part (test study B), the effect of the printing temperature was analysed by comparing specimens printed at 180 ${}^{{}^{\circ}}C$ (considered as the standard printing temperature) and 200 ${}^{{}^{\circ}}C$ denoted as ‘higher temperature’ (HT). An overview of the additively manufactured specimens is provided in Figure 8 HT specimens).

In the test series, for all types and configurations considered seven specimens were printed and tested. This allows a statistical analysis of the results, where mean values, standard deviations and the 95% confidence interval are used for evaluating the results. All specimens/tests are considered in the subsequent presentation of the results, since no specimen failed below a threshold deemed to manipulate a statistical analysis.

## 4. Results and Discussion

Results from uniaxial testing of all types of specimens comprising studies A and B are presented next. First, the stress–strain behaviour of the specimens is analysed where effects of overlap, printing temperature and nozzle diameter size on the mechanical behaviour are evaluated. Subsequently, the characteristic mechanical parameters Young’s modulus (*E*, initial stiffness) and ultimate strength (UTS) are analysed.

Throughout the section the following abbreviations are employed: SF—short fibre reinforcement, LF—long fibre reinforcement, OL—overlap (of 5%) used during printing of the specimens, HT—higher printing temperature used ($200{\;}^{{}^{\circ}}C$). Note that if no ‘OL’ abbreviation is shown, then the standard printing setup has been used without considering overlap. If no ‘HT’ is shown, then the printing temperature determined in the pre-tests of $180{\;}^{{}^{\circ}}C$ has been used.

### 4.1. Stress—Strain Behaviour

#### 4.1.1. General Observations

**Figure 9:**
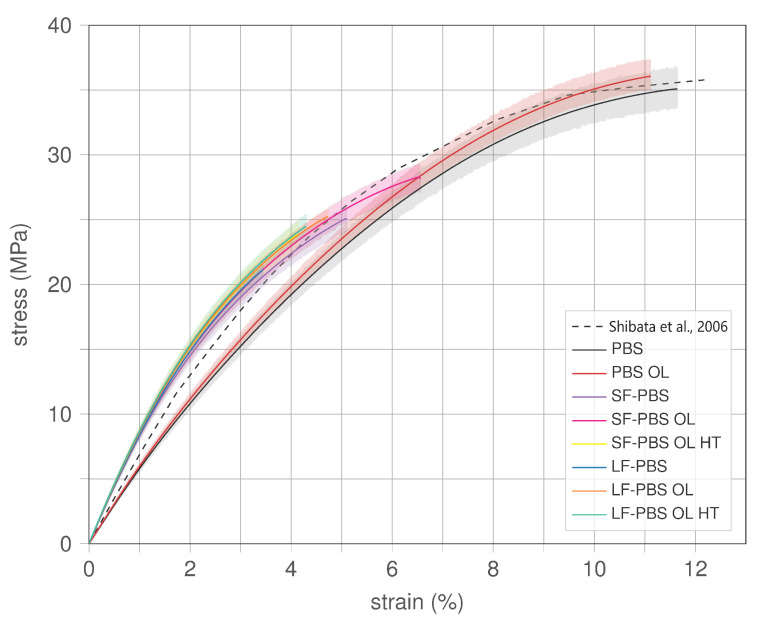
Stress–strain behaviour of PBS and hemp fibre-reinforced PBS; SF—short fibre, LF—long fibre, OL—overlap (5%), HT—higher printing temperature ($200{\;}^{{}^{\circ}}$C instead $180{\;}^{{}^{\circ}}$C) [24].

In Figure 9, the stress–strain response of the uniaxial tests are presented for all types in the form of the mean (thick lines) alongside the respective 95% confidence interval (shaded areas). The mechanical behaviour changes significantly with introducing hemp fibre reinforcement to the PBS. The stress–strain behaviour of (non-reinforced) PBS and PBS OL (black and red lines, respectively) exhibits a softer initial response (smaller stiffness/Young’s Modulus) compared to reinforced PBS (SF-PBS and LF-PBS) but withstands significantly larger stresses (higher ultimate strength) than reinforced PBS. The stress–strain response of PBS is compared to experimental data from [24] (dashed line in Figure 9), where conventionally manufactured PBS was used. Good agreement is observed, where the tensile strength matches the strength of PBS OL (slightly larger than PBS) and the initial tensile modulus is slightly higher for the conventionally manufactured PBS but lower than for the reinforced PBS.

Analysing the responses of the reinforced PBS (SF-PBS and LF-PBS), the stress–strain behaviour is very similar with almost coinciding initial stiffness where LF-PBS specimens show slightly stiffer responses for strains larger than 1%. In contrast, LF-PBS specimens reach smaller ultimate strength in comparison with SF-PBS specimens.

Comparisons of the stress–strain curves of specimens without and with overlap (OL) indicates that implementing such printing strategy improves the mechanical behaviour causing higher stiffness and ultimate strength, which is analysed in detail in Section 4.1.2. The effect of higher printing temperature (specimens labelled with HT) appears to improve the stiffness slightly, but may promote smaller ultimate strength (see Section 4.1.3).

#### 4.1.2. Effect of Overlap

**Figure 10:**
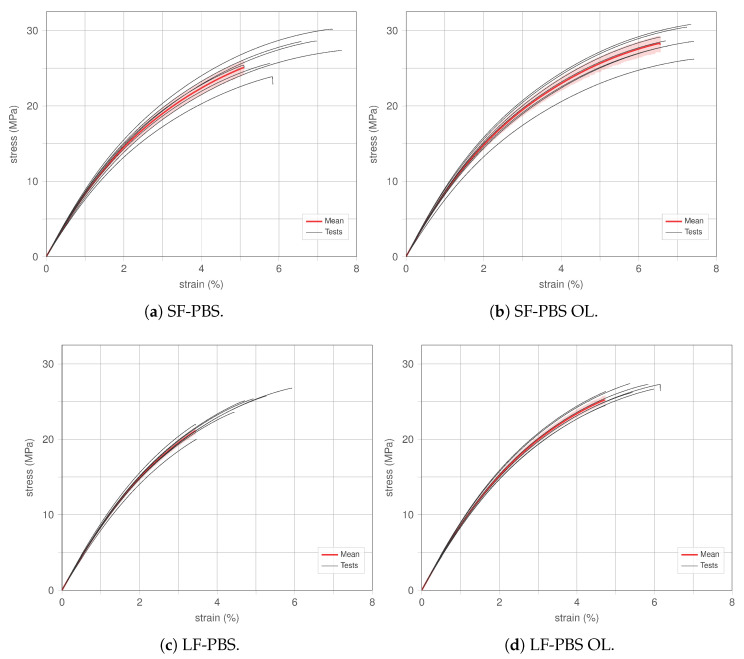
Comparison of the stress–strain behaviour of hemp-reinforced PBS for changing fibre size (SF, LF) and overlap printing parameter (OL).

The beneficial effect of considering an overlap in the printing of (hemp) fibre-reinforced PBS is analysed in detail with the aid of Figure 10. In Figure 10, the stress–strain responses of all tests for specimens with short fibre (SF)- and long fibre (LF)-reinforcement considering ‘no overlap’ and an overlap (OL) of 5% are provided (black thin lines) alongside the corresponding mean responses (red thick lines) with the 95% confidence interval (red shaded areas). It should be noted that mean responses and confidence intervals are only provided up to failure of the specimen exhibiting the lowest ultimate strength to enable a statistical analysis. As a consequence, the maximum stress indicated by the mean curve (also applies to Figure 9) does not correspond to the mean of the ultimate strength of the respective specimen type.

In Figure 10a,b, the effect of overlap for short fibre-reinforced PBS can be analysed. By showing all tests conducted (thin black lines), it can be seen that an overlap (Figure 10b) improves the repeatability and consistency in mechanical responses, while the overall maximum stress and strain values reached considering all tests are very similar between ‘no OL’ and ‘OL’ (max. stress of ca. 30 MPa; max. strain of ca. 7.5%), the scatter for specimens without overlap is significantly larger. Hence, SF-PBS OL exhibits a larger (mean) ultimate strength, which is visualized in Section 4.2. This effect can be associated with the overlap reducing the amount of voids and gaps in the printed specimens, thus promoting improved ultimate strengths.

Similar behaviour can be seen for long fibre-reinforced PBS in Figure 10c,d. Both types of specimens (LF-PBS, Figure 10c; LF-PBS OL, Figure 10d) show similar overall maximum stresses and strains (max. stress of ca. 27 MPa; max. strain of ca. 6%), whilst specimens without overlap suffer from a large scatter in mechanical responses. The effect of improving the (mean) ultimate strength with introducing overlap in the print of the specimens appears to be even more pronounced than for short fibre-reinforced PBS, which is highlighted in Section 4.2.

Regarding the the initial stiffness of the specimens, for both types (SF-PBS and LF-PBS) introducing overlap in the print of the specimens has only a minor effect. Analysing the mean curves (red lines) in Figure 10, marginally larger stresses are associated with strain levels of 1 and 2% when comparing the responses of specimens without and with overlap. This may be related to inserting more material within the defined cross-section by employing overlap. However, it should be noted that micro damaging promoted by the heterogeneity of the material (due to fibre reinforcement) may already be present at such strain levels, where reducing voids and defects with the aid of the overlap reduces such effects. Furthermore, the polarity of PBS and its strong interactions with hemp may exhibit beneficial effects regarding micro-cracking. A comparison of the initial stiffnesses following the standard ASTMD3039 (evaluated at very small strain levels) is provided in Section 4.2.

#### 4.1.3. Effect of Printing Temperature

After determining that an overlap in the print of the specimens improves the mechanical performance, the effect of different printing temperatures is analysed. Therefore, short fibre- and long fibre-reinforced specimens with overlap (SF-PBS OL and LF-PBS OL, respectively) are printed with two temperature: $180{\;}^{{}^{\circ}}$C and $200{\;}^{{}^{\circ}}$C. A temperature of $180{\;}^{{}^{\circ}}$C was determined as the required printing temperature for hemp fibre-reinforced PBS specimens during the pre-test series. Such specimens are labelled as before, thus SF-PBS OL and LF-PBS OL, respectively. Specimens printed with the temperature of $200{\;}^{{}^{\circ}}$C are labelled ‘SF-PBS OL HT’ and ‘LF-PBS OL HT’, respectively.

**Figure 11:**
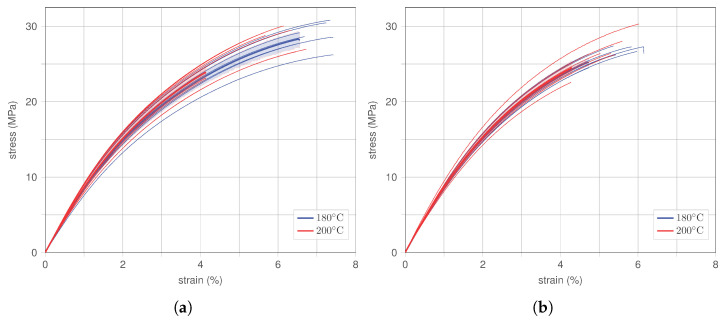
Comparison of stress–strain behaviour of hemp-reinforced PBS for changing fibre size (SF, LF) and printing parameter temperature (HT). (**a**) SF-PBS OL ($180{\;}^{{}^{\circ}}$C) and SF-PBS OL HT ($200{\;}^{{}^{\circ}}$C). (**b**) LF-PBS OL ($180{\;}^{{}^{\circ}}$C) and LF-PBS OL HT ($200{\;}^{{}^{\circ}}$C).

In Figure 11, the stress–strain behaviour for both types (SF-PBS in Figure 11a and LF-PBS in Figure 11b) are provided, where responses of all specimens alongside the respective mean and confidence interval are shown. Specimens printed at $180{\;}^{{}^{\circ}}$C and $200{\;}^{{}^{\circ}}$C are illustrated in blue and red, respectively.

For both types (SF-PBS and LF-PBS), a printing temperature of $200{\;}^{{}^{\circ}}$C causes a slightly stiffer response compared to specimens printed at $180{\;}^{{}^{\circ}}$C. Such behaviour may be associated with PBS having a lower viscosity at $200{\;}^{{}^{\circ}}$C further decreasing voids and defects during the print. However, a minor decrease in ultimate strength can also be observed for specimens with higher printing temperature, which is quantified in Section 4.2. It should be stressed that such decrease lies within statistical variations considering the number of specimens tested per type (see single specimens failing at significantly smaller stresses for $200{\;}^{{}^{\circ}}$C in Figure 11).

#### 4.1.4. Effect of Nozzle Size

**Figure 12:**
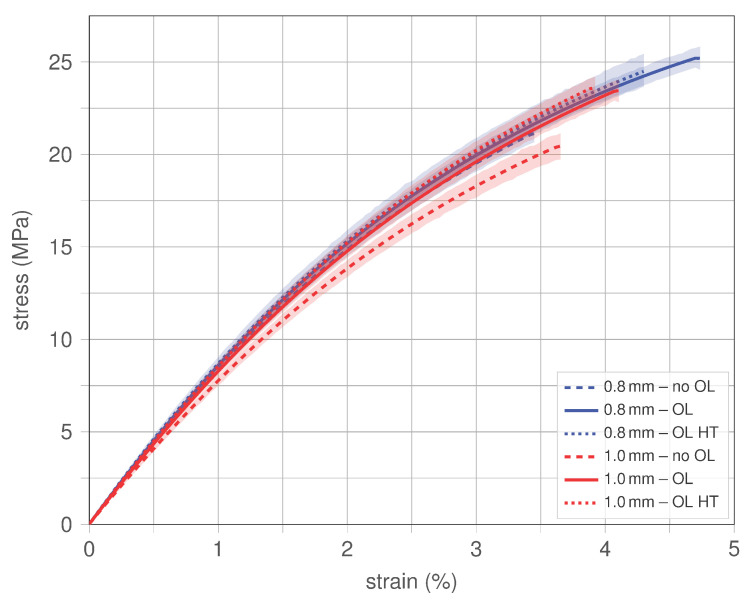
Stress–strain behaviour comparison of nozzle sizes 0.8 mm and 1.0 mm.

Since printing LF-PBS specimens with a 0.8 mm nozzle causes several problems (e.g., frequent clogging of the nozzle), all LF-PBS specimens were also printed using a 1.0 mm nozzle. The resulting stress–strain responses for all types (LF-PBS, LF-PBS OL and LF-PBS OL HT) are compared in Figure 12. The results are presented in the form of the mean with the 95% confidence interval (up to failure of the first specimen). Responses associated with nozzle sizes of 0.8 mm and 1.0 mm are shown in blue and red, respectively. Curves corresponding to LF-PBS (without OL), LF-PBS OL and LF-PBS OL HT are illustrated in the form of dashed, solid and dotted lines, respectively.

Figure 12 visualizes that irrespective from the nozzle geometry specimens without overlap (no OL) exhibit the worst mechanical performance in terms of strength and stiffness (see dashed lines), which also relates to the large scatter observed when analysing the effect of overlap (*cf.* Figure 10). It should be noted that all other types show very similar behaviour where the stress–strain responses are almost coinciding. It appears that the smaller nozzle size (0.8 mm) improves the repeatability, thus exhibiting the smallest scatter in the general behaviour and ultimate strength which causes the mean responses (blue lines) shown in Figure 12 to reach higher ultimate strengths. This can be related to a more precise print using the smaller nozzle size exhibiting less deviations in the final geometry and less defects and voids. For both nozzles, a higher printing temperature (HT) results in a minor decrease in non-linearity of the stress–strain response causing slightly higher stresses at same applied strain levels, which becomes apparent for strains larger than 1%. As discussed before, despite exhibiting an almost coinciding response, the smaller nozzle appears to yield more consistent specimens, underlined by the higher ultimate strength reached in Figure 12. Note that this value represents the lowest strength reached by any specimen of the respective type (*cf.* Figure 10). The mean of ultimate strength and stiffness are presented next.

### 4.2. Stiffness and Strength

The initial stiffness (Young’s modulus *E*) and the ultimate strength of all types of specimens are analysed. Mean values are considered. The stiffness is determined in accordance with standard ASTMD 39039 [33]. For long fibre-reinforced specimens, the analysis is limited to specimens printed with the 0.8 mm diameter nozzle, since the larger nozzle diameter (1.0 mm) resulted in very similar behaviour with slightly less strong specimens.

**Figure 13:**
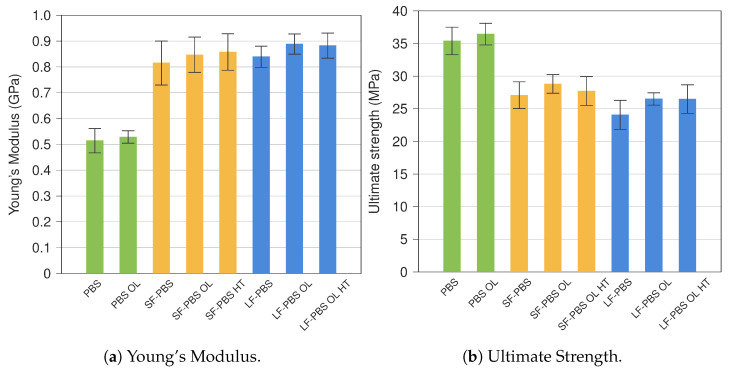
Overview of characteristic material parameters for all types tested.

Both material parameters are presented in Figure 13 in the form of bar charts: Young’s modulus in Figure 13a and ultimate strength in Figure 13b. Results for ‘pure’ PBS, short fibre- and long fibre-reinforced PBS are shown in green, yellow and blue, respectively, where the common abbreviations are employed (SF—short fibre, LF—long fibre, OL—overlap, HT—higher temperature). Standard deviations are also added to each result.

In Figure 13a, it can be seen that hemp fibre reinforcement significantly increases the stiffness of the material, which relates to hemp having a larger Young’s modulus than PBS. An increase of approximately 59 and 63% was obtained comparing PBS with SF-PBS and LF-PBS, respectively. Moreover, the stiffness also increases by introducing overlap (OL), where an increase of 4 and 6% was determined comparing ‘no OL’ with OL. Note that pure PBS also shows a minor increase in stiffness (3%) when using overlap in the additive manufacturing of the specimens. It should be noted that the Young’s modulus of pure PBS is within the range documented in Table 1 highlighting the quality of the additive manufacturing. In contrast, a higher printing temperature does not affect the Young’s modulus, where negligible deviations were observed comparing ‘OL’ with ’OL HT’. Overall, it should be noted that short fibre- and long fibre-reinforced PBS possess very similar stiffnesses with only slightly higher measures for LF-PBS. Quantitatively, a maximum (mean) stiffness of 890 MPa could be obtained by reinforcing PBS with hemp (LF PBS OL), which is roughly 60% of the upper bound stiffness calculated in Section 2.3. Considering the limited length as well as the arbitrary distribution and alignment of the hemp fibres, this measure appears plausible.

The reverse relationship is present analysing the ultimate strength of the specimens, where pure PBS clearly exhibits the highest ultimate strength. SF-PBS and LF-PBS have an approximately 23 and 32% smaller ultimate strength than pure PBS, respectively. A similar trend is documented in [20]. It should be stressed that overlap improves the ultimate strength for all specimens types, where increases of 3, 6 and 10% were determined for PBS, SF-PBS and LF-PBS, respectively. As for the initial stiffness, no further improvement could be achieved with the aid of a higher printing temperature, where SF-PBS even indicates a decline in strength. Note that the standard deviation is the largest for specimens printed at higher temperature (HT). In contrast, specimens with overlap (OL) show for all specimen types the smallest standard deviation. This underlines that using overlap in the printing process reduces the vulnerability to voids and defects and thus results in specimens that exhibit improved mechanical characteristics with a small scatter in their stress–strain response. It is also noteworthy that, despite having very similar Young’s moduli, there is a considerable decrease in strength comparing SF-PBS with LF-PBS, where LF-PBS possesses an approximately 10 and 9% lower ultimate strength for prints without and with (OL) overlap, respectively.

## 5. Conclusions

The current study aims at making contributions towards the exploration of sustainable material concepts by reinforcing the biodegradable polymer PBS with the natural fibre material hemp. The work comprised two parts: first, determining parameters that enable the additive manufacturing of hemp fibre-reinforced PBS; second, studying the mechanical behaviour of hemp fibre-reinforced PBS by means of an experimental test series. The latter comprises the analysis of the effect of varying printing parameters such as the printing temperature and using an overlap.

Three material (specimen) types are considered: pure PBS, short hemp fibre-reinforced PBS (length ≤ 2 mm) and long hemp fibre-reinforced PBS (length ≤ 10 mm). In addition, specimens with two different overlap configurations: ‘no overlap’ and an overlap of 5% (‘OL’), as well as two printing temperatures: 180 ${}^{{}^{\circ}}C$ and 200 ${}^{{}^{\circ}}C$ (‘HT’), are additively manufactured, tested and analysed. The material responses from uniaxial material testing are analysed regarding the general characteristics of the stress–strain behaviour, the initial stiffness (Young’s modulus) and the ultimate strength. Good agreement with experimental data from the literature was obtained for ’pure’ PBS. The test series has highlighted that overlap improves the mechanical behaviour of additively manufactured specimens, being specifically pronounced for hemp fibre-reinforced specimens where the stiffness and strength increases by up to 6 and 10%, respectively, comparing ‘no overlap’ to a 5% overlap. In contrast, higher printing temperatures have not resulted in a statistically relevant improvement in the mechanical response of the additively manufactured specimens.

Introducing hemp fibre-reinforcement significantly increased the stiffness of the material, where up to 63% larger values have been determined comparing pure PBS to reinforced PBS. On the other hand, reductions in strength occur by reinforcing PBS with hemp fibres. It should be noted that, while exhibiting very similar stiffnesses, short fibre-reinforced PBS is less affected in terms of reducing strength (by 23%) than long fibre PBS (by 32%). In addition, the additive manufacturing of short fibre-reinforced PBS is less prone to obstacles such as nozzle clogging than long fibre-reinforced PBS. Based on the study performed, short fibre-reinforced PBS employing a 5% overlap and manufactured at 180 ${}^{{}^{\circ}}C$ appears to be the optimal choice regarding printability and mechanical performance, whenever the reduction in ultimate strength compared to pure PBS can be accepted. The identified printing parameters (180 ${}^{{}^{\circ}}C$ and 5% overlap) enable reliable prints of hemp fibre-reinforced PBS which is underlined by the low scatter obtained in the mechanical response of such specimens, highlighted by small standard deviations and narrow bands of the 95% confidence interval employed during the evaluation of the experimental test series. It is noteworthy that further capabilities for improving the mechanical response (stiffness and strength) can be achieved by an improved alignment of the hemp fibre particles in the filament, where currently fibres are randomly distributed.

## Figures and Tables

**Table 1:** Material properties of PBS and hemp fibre [23,25].

Material	Tensile Modulus (MPa)	Tensile Failure Stress (MPa)	Tensile Failure Strain (%)	Density g/cm³
PBS [22,31]	300–707	20–40	15–170	1.26
Hemp fibre [14]	17.6–66	310–886	2–4	1.4–1.6

**Table 2:** Extrusion parameter settings for homogeneous biocomposite microstructures and filament dimensions.

Extrusion Parameters	Screw Speed (rpm)	Temperature Zone (°C)				
		1	2	3	4	5
Twin-screw	100	50	120	105	105	100
Single-screw	20	50	100	120	105	100

**Table 3:** Printing parameters according to the slicer Cura.

Specimen Geometry	
Length $L_{1}$	120 mm
Length $L_{2}$	30 mm
Width *W*	15 mm
Height *H*	2 mm
**FFF 3D printer**	
Printer	PRUSA i3 MK3S+
Nozzle Diameter	0.8 mm and 1.0 mm (1.0 mm for LF-PBS)
**Quality**	
Layer Height	0.4 mm
Initial Layer Height	0.4 mm
Line Width	0.4 mm
**Walls**	
Wall Line Count	0
**Top/Bottom**	
Top Layers	0
**Infill**	
Infill Density	100%
Infill Line Distance	0.4 mm and 0.38 mm (0% and 5% overlap)
Infill Pattern	Lines
Infill Line Directions	[90]
Infill Wipe Distance	0.1 mm
**Material**	
Printing Temperature	180.0 °C and 200.0 °C
Build Plate Temperature	85.0 °C
Flow	100.0%
**Speed**	
Print Speed	35.0 mm/s
Travel Speed	120.0 mm/s
z Hop Speed	10.0 mm/s
Number of Slower Layers	0
**travel**	
Enable Retraction	on
Retraction Distance	0.8 mm
Retraction Speed	35.0 mm/s
Avoid Printed Parts When Travelling	on
Z Hop When Retracted	on
Z Hop Height	1.0 mm
**Cooling**	
Enable Print Cooling	off

**Table 4:** Adjustments of the printing parameters of the pilot investigations.

Specimen Geometry (pilot investigations)	
Length $L_{1}$	60 mm
Length $L_{2}$	0–30 mm
Width *W*	10 mm
Height *H*	2 mm
**FFF 3D printer**	
Printer	PRUSA i3 MK3S+
Nozzle Diameter	0.8 mm and 1.0 mm
**Quality**	
Layer Height	0.3–0.4 mm
Initial Layer Height	0.2–0.4 mm
Line Width	0.4 mm
**Walls**	
Wall Line Count	0–2
**Top/Bottom**	
Top Layers	0–2
**Infill**	
Infill Line Distance	0.4–0.32 mm (0–20% overlap)
Infill Line Directions	[0] and [90]
**Material**	
Printing Temperature	175.0–200.0 °C
Build Plate Temperature	60.0 °C
**Speed**	
Print Speed	20.0–60.0 mm/s
**Travel**	
Avoid Printed Parts When Travelling	on and off
**Cooling**	
Enable Print Cooling	on and off
